# Case report: Rare persistent complete donor chimerism and GVHD following micro-transplantation from HLA haplotype homozygous donors

**DOI:** 10.3389/fimmu.2022.1005364

**Published:** 2022-09-15

**Authors:** Lingling Liu, Qingya Cui, Mengyun Li, Zheng Li, Sifan Chen, Yunju Ma, Jun He, Depei Wu, Xiaowen Tang

**Affiliations:** ^1^ National Clinical Research Center for Hematologic Diseases, Jiangsu Institute of Hematology, The First Affiliated Hospital of Soochow University, Suzhou, China; ^2^ Institute of Blood and Marrow Transplantation, Collaborative Innovation Center of Hematology, Soochow University, Suzhou, China; ^3^ Department of HLA Laboratory, Jiangsu Institute of Hematology, The First Affiliated Hospital of Soochow University, Suzhou, China

**Keywords:** acute myeloid leukemia, micro-transplantation, HLA haplotype homozygous donor, complete donor chimerism, graft-versus-host disease (GVHD)

## Abstract

HLA-mismatched hematopoietic stem cell micro-transplantation (MST) is an effective treatment for older patients (≥60 years) with acute myeloid leukemia. Donor selection for MST is broad, ranging from HLA fully mismatched unrelated donors to HLA partially matched related donors. However, the influence of HLA haplotype homozygous donors such donors on MST has not been studied. Such donors has been reported to be associated with a higher risk of graft-versus-host disease (GVHD) in transfusion and cord blood transplantation (CBT). Additionally, sustained complete donor chimerism is rare in MST and usually accompanied by severe acute GVHD and death. Herein, we report the first case of MST using an HLA haplotype homozygous donor. The patient developed persistent complete donor chimerism (donor cells>95%) for 7 months and prolonged isolated thrombocytopenia (PT) for 3 months, after receiving MST from his HLA homozygous son. Grade I acute GVHD presented on day 12 post-MST and it was controlled by timely immunosuppressive treatment. Then he maintained complete molecular remission, complete donor chimerism and mild GVHD for 5 months. However, moderate overlapping GVHD with skin, oral, eyes, and intestinal involvement developed after he self-discontinued Tacrolimus treatment. Fortunately, the GVHD was controlled after intensive anti-rejection therapy and Tacrolimus is now being continued for prophylaxis. This case underscores that HLA haplotype homozygous donors might not be a good choice for MST and GVHD prophylactic should be administrated if such donors have to be selected.

## Introduction

Although allogeneic hematopoietic stem cell transplantation (allo-HSCT) is a potentially curative therapy for acute myeloid leukemia (AML), older patients (≥60 years) are susceptible to transplant-related toxicity and severe graft-versus-host disease (GVHD) (1). Infusion of HLA-mismatched donor granulocyte colony-stimulating factor (G-CSF) mobilized peripheral blood stem cells (G-PBSCs) combined with chemotherapy (micro- transplantation [MST]) is capable of producing donor micro-chimerism (donor cells<1%), improving survival, and precluding GVHD (2). It’s a safe and effective treatment for older patients with AML (2–6).

HLA - mismatched donors are generally selected for MST and the efficacy of unrelated donor-derived MST is similar to or higher than that of related donor-derived MST (2, 3). HLA haplotype homozygous donors (whose HLA is homozygous for one of the recipient’s haplotypes) were reported to cause fatal transfusion-related GVHD when patients receive a transfusion of nonirradiated blood components as early as 1990 (7–9). And such donors were also believed to increase the risk of acute GVHD in cord blood transplantation (CBT) (10). However, the influence of HLA haplotype homozygous donors on MST has not been studied. Additionally, sustained complete donor chimerism is not required as well as rare in MST. It was usually accompanied by severe acute GVHD due to lack of or no response to immunosuppressants (4–6). Herein, we report the first case of MST using an HLA haplotype homozygous donor. The patient developed persistent complete donor chimerism (donor cells>95%) for 7 months and prolonged isolated thrombocytopenia (PT) for 3 months, after receiving MST from his son, whose HLA was homozygous at HLA-A, B, C, DR, and DQ loci. Grade I acute GVHD presented on day 12 post-MST and it was controlled by timely immunosuppressive treatment. Then he maintained complete molecular remission, complete donor chimerism and mild GVHD for 5 months. However, the patient subsequently developed moderate overlapping GVHD with skin, oral, eyes, and intestinal involvement after he self-discontinued Tacrolimus treatment. Fortunately, the GVHD was controlled after intensive anti-rejection therapy and Tacrolimus is now being continued for prophylaxis. This report could provide some important clinical experience for the selection and use of HLA haplotype homozygous donors in MST.

## Case presentation

**Figure 1:**
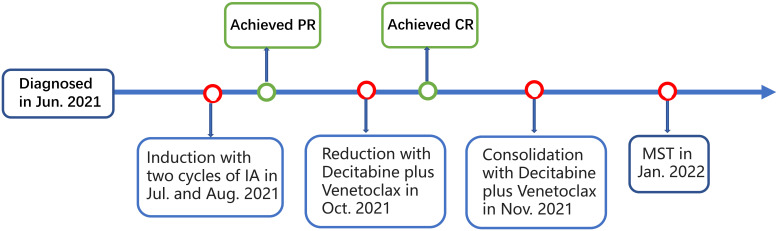
Schematic representation of the patient’s treatment history. IA, idarubicin and cytarabine; PR, partial remission; CR, complete remission; MST, micro-transplantation.

A 62-year-old man was presented with fever and swollen lymph nodes in June 2021 in local hospital. Routine blood tests showed a white blood cell (WBC) count of 1.3 ×10^9^/L, and hemoglobin (Hb) of 82 g/dL. Bone marrow (BM) aspirates showed 65% blasts by histology and 64.8% blasts by flow cytometry that were positive for CD123, CD117, CD13, CD33, HLA-DR, and CD45, and negative for CD34. Cytogenetics demonstrated normal male chromosomes. Next-Generation Sequencing (NGS) revealed mutations of KMT2A-PTD, DNMT3A, IDH2, and STAG2. A diagnosis of AML with KMT2A-PTD (high risk) was established. The patient received two cycles of induction chemotherapy with idarubicin and cytarabine (IA) and achieved partial remission (PR). He then was admitted to our hospital for further treatment. Complete peripheral blood (PB) cell count showed a leukocyte count of1.18 × 10^9^/L, Hb of 75 g/L, and platelets of 64 × 10^9^/L. Blast count of BM was 7% by histology and 6.41% by flow cytometry, blasts were positive for CD117, CD13, CD33, CD38, HLA-DR, and CD45, and negative for CD34, CD19, and CD10. Chromosome analysis showed 46,XY [20]. The mutations of KMT2A-PTD, DNMT3A, IDH2, and STAG2 were reconfirmed by NGS. He then underwent reinduction of decitabine plus venetoclax and achieved complete remission (CR). Minimal residual disease (MRD) based on flow cytometry was negative (<1.1×10^-4^). He then received one cycle of consolidation with the same regimen (**Figure 1**).

**Figure 2:**
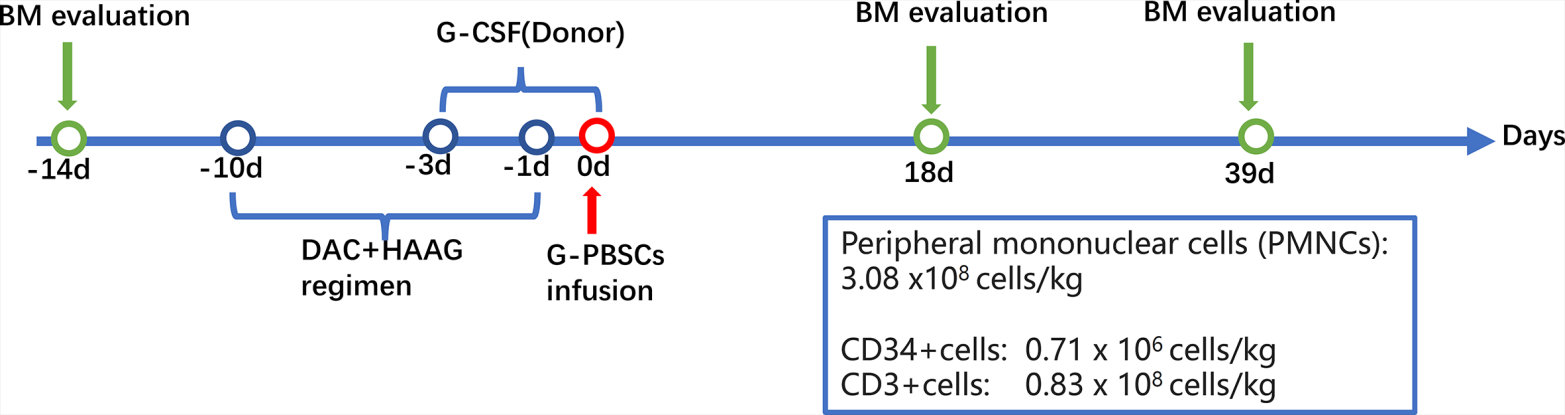
Flowchart of the micro-transplantation protocol. BM, bone marrow; DAC, decitabine; G-CSF, granulocyte colony-stimulating factor; HAAG, homoharringtonine (HHT), cytarabine (Ara-C), aclarubicin (Acla), and G-CSF; MST, micro-transplantation.

However, due to his frail physical condition and lung infections, he was excluded from standard allo-HSCT. Therefore, the patient was subjected to MST for prolonged survival. The patient was pretreated with decitabine plus HAAG regimen (homoharringtonine (HHT), cytarabine (Ara-C), aclarubicin (Acla), and G-CSF) (11). Peripheral blood mononuclear cells (PBMCs) at a dose of 3.08 x10^8^ cells/kg of body weight from his son whose HLA typing was homozygous at HLA-A, B, C, DR, and DQ loci (**Table 1**) were administered at 24 hours after the HAAG regimen (Day0), including 0.71×10^6^cells/kg CD34^+^cells and 0.83×10^8^cells/kg CD3^+^cells. No GVHD prophylactic was administered (**Figure 2**).

**Table 1:** The result of patient and donor HLA typing.

	A*	B*	C*	DRB1*	DQB1*	DPB1*	DQA1*	DPA1*	DRB345*
**patient**	**11:02**	**27:04**	**12:02**	**12:02**	**03:01**	**21:01**	**06:01**	**01:03**	DRB3*03:01
	**33:03**	**58:01**	**03:02**	**03:01**	**02:01**	**04:01**	**05:01**	**01:03**	DRB3*02:02
**donor**	**11:02**	**27:04**	**12:02**	**12:02**	**03:01**	**21:01**	**06:01**	**01:03**	DRB3*03:01
	**11:02**	**27:04**	**12:02**	**12:02**	**03:01**	**02:02**	**06:01**	**02:02**	DRB3*03:01

**Figure 3:**
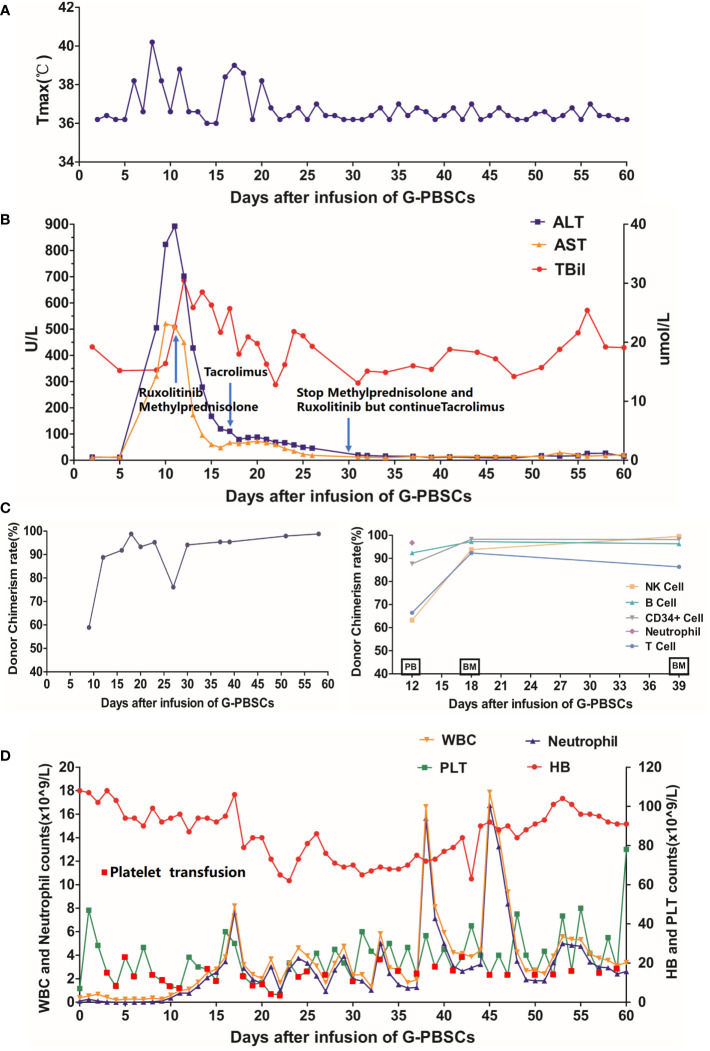
**(A)** Changes in body temperature after the infusion; **(B)** Changes in alanine aminotransferase, aspartate aminotransferase, and total bilirubin after the infusion; **(C)** Changes in hematopoietic donor chimerism of the total PB cells (Left Panel) and sorted PB or BM cells (Right Panel) by polymerase chain reaction-based-short tandem repeats assay with a sensitivity of 1%; **(D)** Changes in PB cell count after the infusion. G-PBSCs, granulocyte colony-stimulating factor-mobilized peripheral blood stem cells; ALT, alanine aminotransferase; AST, aspartate aminotransferase; TBil, total bilirubin; PB, peripheral blood; BM, bone marrow; WBC, white blood cell; HB, hemoglobin; PLT, platelet. PB and BM on the abscissa in **(C)** indicate the source of specimens for this examination.

After the infusion of G-PBSCs, the patient developed a fever on day 6 that lasted for 14 days and peaked at 40.2°C. (**Figure 3A**) Elevated liver enzymes were detected on Day 9 and persisted for approximately 2 weeks (**Figure 3B**). Donor chimerism in PB was 58.9% detected by polymerase chain reaction-based short tandem repeats (PCR-STR) on Day 9, which increased to complete donor chimerism (98.8%) by Day 18 (**Figure 3C**). As there was no evidence of infection, we immediately initiated immunosuppressive therapy (Methylprednisolone, Tacrolimus, and Ruxolitinib) and monitored the chimerism closely to save the patient from severe acute GVHD. On Day 12, PCR-STR of sorted PB cells revealed donor chimerism 87.62%, 63.24%, 66.44%, 96.73%, and 92.35% for CD34^+^stem cells, NK cells, T lymphocytes, Neutrophils, and B lymphocytes, respectively (**Figure 3C**), accompanied by mild rashes on the back, chest, palms. He was diagnosed with grade I acute GVHD according to the modified Glucksberg-Seattle criteria (12). After immunosuppressive and hepatoprotective therapy, the patient’s body temperature returned to normal, the rash disappeared, and the liver enzymes decreased significantly. His neutrophils recovered on Day 18 while platelet engraftment was delayed (**Figure 3D**). His BM aspirate was re-examined on Day 39. Morphology showed no blasts. MRD based on flow cytometry was negative (<7.9 ×10^-5^). The mutations were also not detected by quantitative polymerase chain reaction (qPCR) and PCR-pair end sequencing. PCR-STR of sorted BM cells revealed donor chimerism 98.16% and 86.34% for CD34^+^stem cells and T lymphocytes, respectively (**Figure 3C**).

The patient maintained complete molecular remission, complete donor chimerism (donor cells>95%) and mild GVHD for 5 months. His platelets recovered about 120 days after the infusion of G-PBSCs. However, it’s noteworthy that he developed moderate overlapping GVHD with skin, oral, eyes, and intestinal involvement after he self-discontinued Tacrolimus 5 months post-MST. Then Methylprednisolone, Tacrolimus, Ruxolitinib, and CD25 monoclonal antibody were given and the GVHD was controlled. Now the patient is still in complete molecular remission with no infection, rash, diarrhea, or abnormal liver function. Tacrolimus is now being continued for prophylaxis due to complete donor chimerism.

## Discussion and conclusion

MST is an important treatment for patients who cannot tolerate standard allo-HSCT or lack suitable donors (2–6). Donor selection for MST is broad, ranging from HLA fully mismatched unrelated donors to HLA partly matched related donors and the efficacy of unrelated donor-derived MST is believed to be similar to or higher than that of related donor-derived MST (2, 3). HLA haplotype homozygous donors have been reported to cause fatal transfusion-associated GVHD when patients receive a transfusion of nonirradiated blood components as early as 1990. The study by Morishima et al. (10) also showed that such donors could increase the risk of acute GVHD in CBT (7). However, the effect of HLA haplotype homozygous donors on MST remained unstudied. This report provides the first description of the clinical presentations of using a such donor in MST and as well as some important clinical experience.

According to previous studies, there was almost no persistent complete donor chimerism in MST (4–6). The patient described herein developed stable complete donor chimerism after MST from his HLA homozygous son, despite no prior administration of immunosuppression. That indicated grafts from such donors are less likely to be rejected, and the most important reason for that maybe the absence of major allogeneic HLA antigens in the host-versus-graft direction as described in the transfusion and CBT (7–10). Furthermore, homozygous donor cells would have advantages over the patient’s hematopoietic cells for survival and reimplantation during BM regeneration from chemotherapy-induced hypoplasia, which had been reported by Wong and colleagues (13). Complete donor chimerism was often accompanied by severe acute GVHD in MST (4–6). However, the patient in our case only presented grade I acute GVHD, partially due to the patient having completed only one cycle infusion of G-PBSCs, including very few T cells, coupled with the timely administration of immunosuppressants, thus avoiding severe acute GVHD. However, the patient developed moderate overlapping GVHD with skin, oral, eyes, and intestinal involvement after he self-discontinued Tacrolimus 5 months post-MST. It showed that immediate and continuous GVHD prophylactic was really essential when using HLA haplotype homozygous donors in MST. The number of CD34^+^ cells in the graft is a critical factor in neutrophil and platelet recovery, and the latter appears to be more important (14). This may partially explain the patient’s PT in this case. Additionally, impaired BM microenvironment and the presence of platelet-specific antibodies may also be important factors for this patient (15).

In summary, HLA haplotype homozygous donors could cause unexpected complete donor chimerism and bring a high risk of severe GVHD as well as may result in unfavorable platelet recovery in MST. Therefore, in order to ensure micro-chimerism, avoid severe GVHD and poor graft function, it’s better to exclude HLA haplotype homozygous donors in MST and GVHD prophylactic should be administrated if such donors have to be selected.

## Data availability statement

The original contributions presented in the study are included in the article/supplementary material. Further inquiries can be directed to the corresponding authors.

## Ethics statement

The studies involving human participants were reviewed and approved by the Ethics Committee of the First Affiliated Hospital of Soochow University. The patients/participants provided their written informed consent to participate in this study. Written informed consent was obtained from the individual(s) for the publication of any potentially identifiable images or data included in this article.

## Author contributions

LL was the main writer of the case report, contributing to all of the sections of the report. XT, QC, ML, ZL, and SC provided direct patient care while the patient was hospitalized. XT, QC, and YM helped write part of the case report and edited the case report. JH was in charge of HLA typing. All authors read and approved the final manuscript.

## Acknowledgments

The authors would like to thank all members of the study team, the patient and their families.

## Conflict of interest

The authors declare that the research was conducted in the absence of any commercial or financial relationships that could be construed as a potential conflict of interest.

## Publisher’s note

All claims expressed in this article are solely those of the authors and do not necessarily represent those of their affiliated organizations, or those of the publisher, the editors and the reviewers. Any product that may be evaluated in this article, or claim that may be made by its manufacturer, is not guaranteed or endorsed by the publisher.
